# Optimized Isolation and Characterization of C57BL/6 Mouse Hepatic Stellate Cells

**DOI:** 10.3390/cells11091379

**Published:** 2022-04-19

**Authors:** Alexandre Balaphas, Jeremy Meyer, Cécile Gameiro, Aurélien Frobert, Marie-Noëlle Giraud, Bernhard Egger, Leo H. Bühler, Carmen Gonelle-Gispert

**Affiliations:** 1 Division of Digestive Surgery, University Hospitals of Geneva, 1205 Geneva, Switzerland; alexandre.balaphas@hcuge.ch (A.B.); jeremy.meyer@hcuge.ch (J.M.); 2 Department of Surgery, Clinical Medicine Section, Faculty of Medicine, University of Geneva, 1206 Geneva, Switzerland; 3 Flow Cytometry Core Facility, Faculty of Medicine, University of Geneva, 1206 Geneva, Switzerland; cecile.gameiro@unige.ch; 4 Surgical Research Unit, Faculty of Science and Medicine, Section of Medicine, University of Fribourg, 1700 Fribourg, Switzerland; aurelien.frobert@unifr.ch (A.F.); marie-noelle.giraud@unifr.ch (M.-N.G.); bernhard.egger@unifr.ch (B.E.); leo.buhler@unifr.ch (L.H.B.)

**Keywords:** hepatic stellate cells, MACS, CD11b, autofluorescence, CD38

## Abstract

To obtain meaningful results of hepatic stellate cell (HSC) function, it is crucial to use highly pure HSC populations. Our aim was to optimize HSC isolation from mice livers without exploiting the characteristically transient vitamin A autofluorescence of HSC. HSCs were isolated from C57BL/6 mice using a two-step collagenase digestion and Nycodenz gradient separation followed by CD11b-negative sorting step in order to remove contaminating macrophages and dendritic cells. Isolated cells were analyzed for yield, viability, purity, and potential new markers using immunofluorescence and flow cytometry. We obtained a yield of 350,595 ± 100,773 HSC per mouse liver and a viability of isolated cells of 92.4 ± 3.1%. We observed a low macrophage/dendritic cell contamination of 1.22 ± 0.54%. Using flow cytometry, we demonstrated that CD38 was expressed at the surface of HSC subpopulations and that all expressed intracellular markers specific for HSC in the liver. This isolation method, avoiding fluorescent activated cell sorting (FACS), allowed isolation of HSCs with high purity. Further, flow cytometry analysis suggests that CD38 may be a reliable marker of HSCs and may include subpopulations of HSCs without retinoid droplets.

## 1. Introduction

Hepatic stellate cells (HSCs) are specialized pericyte-like cells, located in the space of Disse, that wrap liver sinusoids [1] and interact with both hepatocytes and liver sinusoidal endothelial cells [2]. HSCs are highly relevant for the pathophysiology of the liver. HSCs sense the presence of injury-related soluble factors [3] and their stimulation induces their transformation from a quiescent to an activated state and further to myofibroblasts, the hallmark of liver fibrosis [4]. Over the last three decades, HSC function has been largely studied in rats using various liver fibrosis models. Further, human and rat HSCs have been isolated from the liver. The first method to isolate HSCs from rat livers was reported by Knook et al. in 1982 using metrizamide density separation cushion [5]. Later, different protocols were proposed for rat and human HSC isolation using density gradient separation [5,6,7,8,9], explant culture of liver biopsies [10,11], and fluorescence-activated cell sorting (FACS) by taking advantage of the autofluorescence of HSCs [12]. Vitamin A stored in HSC lipid droplets can be excited by ultraviolet light between 310 and 350 nm wavelength and emits a blue fluorescence with rapid bleaching [13]. However, all these methods have their own drawbacks. Density gradient separation, although very efficient in the separation of high-lipid-content cells that are floating on dense gradients, has the main disadvantage of the poor purity of the preparation [14]. Further, explant cultures also result in low purity and highly heterogonous HSC populations [2,15]. It is generally admitted that fluorescence-activated cell sorting reduces cell viability; thus, resulting in lower yields. Moreover, HSC sorting based on the autofluorescence of HSCs is dependent on the accumulation of vitamin A and favors the isolation of vitamin A-rich HSC [16]. Vitamin-A poor HSCs seem to constitute a different HSC subpopulation and are not selected by this method [17]. Moreover, such a strategy is limited by the availability of cell sorting devices [16].

With the increasing interest for HSCs, isolation protocols were also elaborated for mice [18,19,20], since mice are more suitable for experimental approaches implying genetic modifications. Particularly, C57BL/6 is an important common mouse strain widely used for genetic modifications but rarely for HSC isolation, as other strains such as Balb/c mice, which offer a HSC yield 10 times higher than C57BL/6 mice [14]. For mouse HSC isolation, existing protocols combine density gradient separation, with autofluorescent cell sorting to increase purity [14]. Indeed, HSC sorting protocols are restricted to cell sorting by violet-induced autofluorescence (AF), as specific surface antigens on HSCs have been poorly characterized. Nycodenz density gradient separation appears to be a reliable method to obtain a high yield of high-lipid content cells [14]. However, macrophages and dendritic cells constitute the principal contaminant in the Nycodenz-based protocol for HSC isolation [21].

Isolated HSCs are usually characterized after plating, through analysis of cell morphology and subcellular structures using transmission electron microscopy [15]. Other methods include bright field light microscopy detection of lipid droplets inside the HSC cytoplasm and fluorescence microscopy detection of vitamin A autofluorescence [15]. Few HSC cytoskeletal and intracellular proteins, depending on the HSC state and origin, can be used to identify HSCs [2,15]. Specific surface antigens for HSCs are not described. Recently, CD38 has been proposed for HSC characterization and was validated in rats [22]. Therefore, we describe here a protocol to enrich low-yield HSC preparations isolated from C57BL/6 with a classical gradient method, by eliminating contaminating macrophages [23] and dendritic cells [24] through magnetic-activated cell sorting (MACS) of CD11b-positive cells; thereby avoiding the violet-excitability of vitamin A and its short-lived autofluorescence. The isolated HSCs were analyzed by flow cytometry for the expression of CD38 as a potential marker to detect them.

## 2. Materials and Methods

### 2.1. Animals

C57BL/6 male mice aged >20 weeks were ordered from Janvier Labs (Le Genest-Saint-Isle, France). All procedures were performed in accordance with protocols approved by the Veterinarian Office of Geneva, Veterinarian Office of Fribourg, the University of Geneva, and the University of Fribourg. Mice were housed under standard conditions at the animal facility core of the University of Geneva and Fribourg.

### 2.2. Materials

Falcon polystyrene adherent Petri dishes, 70 µm cell strainers, flow cytometry tubes, polypropylene conical tubes, and polystyrene tissue culture plates were purchased from Corning inc (Corning, NY, USA). CD11b microbeads for mice and LS columns, a QuadroMACS separator, and a MACS MultiStand platform were obtained from Miltenyi Biotec (Bergisch Gladbach, Germany). Chamber slides were from Thermo Fisher Scientific (Waltham, MA, USA). Leica DFC320, Leica DMIL (Leica, Wetzlar, Germany), and Nikon Eclispe TS2 (Nikon, Tokyo, Japan) microscopes and were used for standard light or immunofluorescence analysis. Flow cytometry experiments and cell sorting were performed, respectively, on a BD LSRFortessa or FACSAria (Becton Dickinson, Franklin Lakes, NJ, USA). The centrifuge was a Rotixa 50 RS (Hettich Zentrifugen, Tuttlingen, Germany) and a Multifuge X1R (Thermo Fisher scientific, Waltham, MA, USA).

### 2.3. Reagents

For liver digestion, we used Collagenase type 4 (270 units/mg and 290 units/mg) (Worthington Biochemical Corporation, Lakewood, CA, USA), DNase type 1 (2000 units/mg) (Roche Diagnostics, Risch, Switzerland), Heparin (Drossapharm, Arlesheim, Switzerland), and 40% glucose (Grosse Apotheke Dr. G. Bichsel AG, Interlaken, Switzerland). Culture grade powders for Grey’s Balanced Salt Solution (GBSS) with or without NaCl were from Sigma-Aldrich (Buchs, Switzerland). Nycodenz density gradient solution was from Axon lab AG (Le Mont-sur-Lausanne, Switzerland). William’s E medium with Glutamax, Calcium-free Dulbecco’s phosphate buffered saline (DPBS), Hank’s hank balanced salt solution without calcium, Iscove’s Modified Dulbecco’s Medium (IMDM) with Glutamax supplement, HEPES buffer solution, fetal bovine serum (FBS), penicillin-streptomycin, trypsin, and EDTA were purchased from Life Technologies (Grand Island, USA). Oil-Red-O solution was obtained from Sigma-Aldrich. Fixation buffer was purchased from Biolegend (San Diego, CA, USA). Perm/Wash was obtained from Becton Dickinson. Paraformaldehyde solution for cell fixation was acquired from Sigma-Aldrich. Custom wash solution, STOP solution, and MACS buffer were identical to our previous report [25]. Recombinant human TGF-β1 was obtained from PreproTech (Rocky Hill, CT, USA).

### 2.4. Quantitative Polymerase Chain Reaction

RNA extraction was performed using a kit purchased from Qiagen (Venlo, The Netherlands). RNA quantity and quality were assessed by Agilent 2100 Bioanalyzer (Agilent, Santa Clara, CA, USA). RNA was first reverse transcribed with PrimeScript RT TAKARA (Clontech) using 1.8 ng of RNA. (Mountain View, CA, USA). Then, cDNA was pre-amplified using a Taqman preAmp master kit from Thermo Fisher Scientific. Quantitative real-time polymerase chain reactions were run by the University of Geneva Genomic Platform. The following primers were used: AGCCTGAAAGGACTGATTAATTTGTC, GCTGTTGAAGCTCAATCCCAG for DCN, GCAGGAGCAGAAGACTCAGAATC, CAGTTCTGCGCGATCAGCT for CLE4F, TCCACTTGGTCGCTTTGCT for EEF1A1, TCCATGACAACTTTGGCATTG for GAPDH and GCTCGAGATGTCATGAAGGAGAT for HPRT. EEF1A1, GAPDH, and HPRT were used to normalize RNA quantities.

### 2.5. Antibodies

Monoclonal BV650-conjugated anti-mouse CD45, BD Horizon BUV395-conjugated anti-mouse F4/80 were purchased from Becton Dickinson. Phycoerythrin-cyanine7-conjugated anti-mouse CD38, phycoerythrin-conjugated anti-mouse Nestin, Alexa Fluor 488-conjugated anti-mouse glial fibrillary acidic protein (GFAP), phycoerythrin-cyanine7-conjugated rat IgG2aκ isotype control, phycoerythrin-conjugated mouse IgG2a isotype control, and DRAQ7 were obtained from Thermo Fisher Scientific. Alexa Fluor 488-conjugated rat IgG2aκ isotype and rat anti-mouse F4/80 were acquired from Biolegend. Unconjugated anti-mouse α-smooth muscle actin (α-SMA) was a gift from Sophie Clement (University of Geneva, Geneva, Switzerland). Alexa Fluor 555-conjugated anti-rabbit and Alexa Fluor 488-conjugated anti-rat antibodies were purchased from Invitrogen (Carlsbad, CA, USA). AF488 with Hoechst was obtained from Thermo Fisher Scientific.

### 2.6. Data Analysis

Descriptive statistics were reported as mean ± standard deviation or standard error of the mean. GaphPad Prism version 8 was used to generate statistical analyses and graphical representations (GraphPad Software Inc., La Jolla, CA, USA). BD FACSDiva or BD FlowJo software were used for flow cytometry data analysis and graphical representations (Becton Dickinson) using contour plots with outliers or dot plots.

#### 2.6.1. Liver Perfusion and Digestion

Four to six mice were used for each HSC isolation procedure. Mouse liver two-stage perfusion and digestion steps were similar to the method we have previously reported for liver sinusoidal endothelial cell isolation [25]. After buprenorphine injection and under isoflurane anesthesia, midline laparotomy and thoracotomy incisions, followed by bilateral subcostal incisions, were performed to expose the liver and the heart. Mice were exsanguinated by heart puncture and a perfusion cannula was introduced into the inferior vena cava through the right atrium with a 22-gauge catheter. Liver was perfused at a rate of 5 mL/min, first with washing solution (Hank’s hank balanced salt solution without calcium with HEPES buffer, penicillin–streptomycin, EGTA, glucose and high molecular weight heparin) for 5 min and then with the digestion solution (William’s E with collagenase type 4 and DNase type I) for 6 min. The portal vein was cut immediately when perfusion started for the outflow. Progression of digestion, i.e., advancing softness of liver tissue, was controlled with a delicate pressure on the liver. At the end of digestion, the liver was carefully extracted and manually disrupted with a scalpel on a Petri dish in a collagenase inactivating solution (William’s E with 5% FBS and 1% penicillin–streptomycin) [25]. Each digested liver was collected in 25 mL of inactivating solution and kept on ice.

#### 2.6.2. Isolation of Lipid-Containing Cells

**Figure 1:**
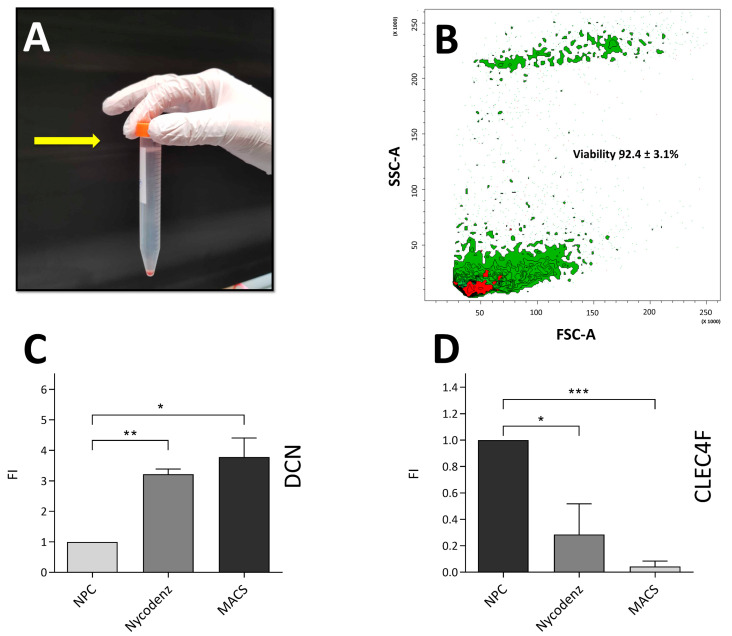
(**A**) After Nycodenz separation, high-lipid-content cells float (white ring) between the Nycodenz solution and the GBSS + NaCl solution (yellow arrow). (**B**) Cell viability assessed with fixable EF 780 viability dye back gated in side scatter (SSC) and forward scatter (FSC)-contour plot with outliers as dots. Green: viable cells; red: dead cells. Proportion expressed as a mean ± SD, *n* = 3. (**C**,**D**) Quantitative polymerase chain reaction assessing DCN (coding for Decorin) (**C**) and CLEC4F (coding for Clefc4f) (**D**) gene expression in cell populations after the two major steps of HSC isolation (Nycodenz and MACS sorting). Values obtained were expressed as fold-increase (FI) where the value obtained by non-parenchymal cell (NPC) population was set as 1. Decorin has been described to be specific to HSCs whereas CLEC4F is expressed by Kupffer cells. * *p* ≤ 0.05, ** *p* ≤ 0.01, *** *p* ≤ 0.00, if not specified, non-significant. *n* = 2.

All centrifugations were performed at 4 °C. Non-digested liver tissue was removed by filtration using a 70 μm cell strainer. Cell suspensions were centrifuged at 68× *g* for 5 min to remove hepatocytes and bile duct cells. Remaining supernatants were then centrifuged at 600× *g* for 10 min to collect non-parenchymal cells. Cell pellets were then resuspended in cold GBSS-NaCl containing 13.2 µg/mL type I DNase and centrifuged at 600× *g* for 10 min. The obtained cell pellets were resuspended in cold GBSS + NaCl and gently mixed with a Nycodenz solution (diluted in GBBS-NaCl) to obtain a final Nycodenz concentration of 8.86%. The suspensions were overlaid carefully with 1.5 mL GBSS + NaCl and centrifuged at 1980× *g* for 15 min. Cells at the interface of the Nycodenz solution and the GBSS + NaCl were harvested and collected in a 50 mL Falcon tube (Figure 1A).

#### 2.6.3. Hepatic Stellate Cells Enrichment Using Magnetic Activated Cell Sorting and Long-Term Selective Adherence

Cells recovered from two Nycodenz suspensions were pooled and suspended in 40 mL MACS buffer and centrifuged at 690× *g* for 15 min. The resulting cell pellet was suspended in 120 μL MACS buffer containing 60 μL CD11b microbeads and incubated for 15 min at 4 °C. Then, unbound CD11b microbeads were removed by adding 2 mL of MACS buffer and centrifugation for 10 min at 300× *g*. The supernatant was removed, and the labeled cells suspended into 2 mL of MACS buffer. The MACS column was rinsed with 3 mL MACS buffer prior to the passage of cell suspension. The flow-through, containing purified unlabeled CD11b- cells (HSC), was collected. CD11+ cells (macrophages and dendritic cells) were also collected by column purge with 4 mL MACS buffer. Both suspensions were centrifuged at 600× *g* for 10 min and their pellet finally suspended in HSC culture medium (IMDM 10% FBS). Cells were counted in a Neubauer chamber and cells were plated in Falcon culture plates at a density of 100,000 isolated cells/W, in 96 W or 250,000 isolated cells/W in 48W. Non-adherent cells were removed from culture after 12 and 18 h by rinsing with DPBS and HSC culture medium change. Cells were then used for immunofluorescence or flow cytometry assays. Cells were not cultured more than one week.

#### 2.6.4. Characterization of Hepatic Stellate Cells Using Immunofluorescence and Oil-Red-O Staining

For α-SMA staining, 25,000 cells/microdrop were cultured on 35 mm Petri dishes during 24 h and then treated by adding TGF-β (final concentration 50 ng/mL) during 7 days. To evaluate the proportion of contaminating macrophages, 25,000 cells/microdrop were seeded on a chamber slide, cultured for 48 h, and stained against F4/80.

For immunofluorescence staining, cells were fixed with paraformaldehyde and permeabilized with 0.2% Triton-X-100 for 15 min and nonspecific sites blocked with PBS/3% BSA/10% goat serum for 1 h. Cells were then incubated with primary antibodies overnight followed by incubation with secondary antibody for 2 h. The nuclei were stained with Hoechst.

For Oil-Red-O staining, 100,000 cells were cultured for 12 h in 96 W plates and fixed with paraformaldehyde. Cells were then incubated for 30 s with Oil-Red-O solution.

#### 2.6.5. Characterization of Hepatic Stellate Cells Using Flow Cytometry

A total of 50,000–100,000 cells were used for each condition. DPBS-washed cells were incubated with DPBS diluted eBioscience™ Fixable Viability Dye eFluor™ 780 or DRAQ7 for 30 min. Then, cells were washed once with DPBS and suspended with flow cytometry buffer (DPBS 2% FBS). After Fc-receptor blocking with anti-mouse CD16/32 for 10 min, conjugated antibodies or their isotypes against surface proteins were added: Brilliant™ Violet 650-conjugated anti-mouse CD45, BD Horizon™, BUV395-conjugated anti-mouse F4/80, phycoerythrin and cyanine Cy5-conjugated anti-mouse CD38, for 20 min. After incubation, cells were washed and fixed with fixation buffer for 30 min. Then, cell pellets were washed and permeabilized with Perm/Wash solution for 20 min and incubated with the following intracellular antibodies and their isotypes for 30 min: phycoerythrin-conjugated anti-mouse Nestin, Alexa Fluor 488-conjugated anti-mouse GFAP. Finally, cells were washed with Perm/Wash solution and suspended in DPBS. Compensations were performed for each channel using VersaComp antibody capture beads (Beckman Coulter, Brea, CA, USA).

## 3. Results

### 3.1. Hepatic Stellate Cells Isolated Using Nycodenz Separation Gradient and Magnetic Activated Cell Sorting

**Figure 2:**
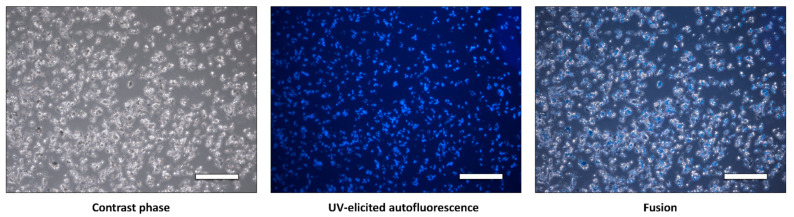
Cytological observation of isolated hepatic stellate cells of HSCs a few hours after plating: contrast phase (lipid droplets are white relative to dark cytoplasm), UV-elicited autofluorescence (blue) and fusion of the first two images. White bar: 200 µm.

A complete HSC isolation protocol was built and tuned after 13 iterations. After Nycodenz gradient separation of high-lipid-content cells and negative selection for contaminating macrophages and dendritic cells with CD11b beads and MACS, we obtained 350,595 ± 100,773 cells per mouse (Figure 1A, Table 1). Viability was assessed using Trypan blue staining (not shown) and was consistent with viability values obtained using flow cytometry which was 92.4 ± 3.1% (Figure 1B). On the other hand, viability assessed directly after FACS for AF was 88.3 ± 5.3%. Quantitative polymerase chain reaction confirmed the presence of HSC-specific expression of Decorin in isolated cell preparations, (Figure 1C). Further, MACS allowed to reduce the presence of CLEC4F expressing cells, a hallmark of Kupffer cells (Figure 1D). The presence of vitamin A droplets in the isolated HSCs was qualitatively determined by observation of the autofluorescence of vitamin A (retinol) (Figure 2). Autofluorescence was rapidly fading and lasted only a few seconds, which is characteristic of vitamin A degradation. Moreover, autofluorescence decreased over time after cell plating (not shown). Additionally, bright field phase contrast light microscopy observation of the cytoplasm clearly demonstrated numerous lipid droplets (Figure S1A), confirmed by Oil Red O staining (Supplementary Figure S1C,D). Remaining macrophage contamination was evaluated qualitatively with F4/80 immunofluorescence (Supplementary Figure S1B).

We further evaluated the ability of HSCs to evolve into α-SMA-expressing myofibroblasts following HSCs culture with TGF-β. As shown, 7 days after TGF-β treatment, all cultured HSCs expressed α-SMA (Figure S2A).

### 3.2. Hepatic Stellate Cell Characterization with Flow Cytometry

The choice of markers for flow cytometry analysis of the isolated HSC population was decided after evaluation and thorough examination of markers and consensus between the co-authors. We excluded surface markers that were described to also be potentially expressed by macrophages, i.a., PDGFR [26] or CD146 [27] (expressed in foam cells). Among the described markers, the glycoprotein CD38 seemed interesting as its expression on HSC can be discriminated from its expression on lymphocytes, natural killer cells, and recruited macrophages (not expressed on residual macrophages) by CD45 expression [28]. To characterize the purity of the isolated HSC population, we also analyzed the expression of intracellular proteins specific for HSCs, such as GFAP and Nestin in the HSC population.

**Figure 3:**
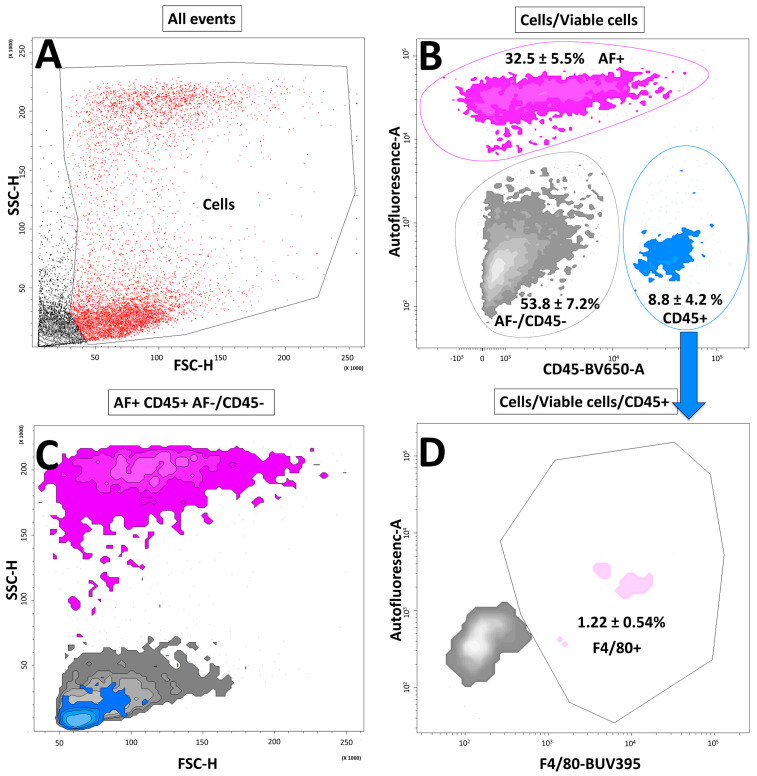
Flow cytometry characterization of isolated hepatic stellate cells. (**A**) Cell morphology allowing to gate cell populations. SSC: side scatter; FSC: forward scatter. (**B**) Selected cells/viable cells (see Figure 1B) gates were analyzed for their expression of CD45 (blue) and the presence of violet-induced autofluorescence (AF) (violet). CD45-/AF- cells are represented in gray. Proportions are expressed as a mean ± SD, *n* = 3. (**C**) Backgating analysis of the cell population morphology: AF+ cells (violet), CD45+ cells (blue), CD45-/AF- cells (gray). SSC: side scatter,;FSC: forward scatter. (**D**) Expression of F4/80 (pink) among the CD45+ population. Proportions are expressed as a mean ± SD, *n* = 3.

Cells were gated for their morphology, removing cell debris, and for viability (Figure 3A,B). Plotting viable cells for violet-induced autofluorescence (AF) and expression of CD45 allowed to distinguish three populations: AF+ cells (mean ± SD of 32.5 ± 5.5%), CD45+ cells (mean ± SD of 8.8 ± 4.2%), and CD45-/AF- cells (mean ± SD of 53.8 ± 7.2%) (Figure 3B). AF+ cells showed a high granularity on side scatter and CD45+ cells constituted a partially homogenous population of small cells (Figure 3C). As HSC autofluorescence contaminated the F4/80 channel, we had to select the CD45+ population to assess F4/80 positivity (Figure 3D). Macrophage contamination measured by flow cytometry was constant with the rough evaluation of contamination performed with immunofluorescence staining and was accounting for 17.4 ± 11.4% of CD45+ viable cells or for 1.22 ± 0.54% of all viable cells analyzed. Therefore, the calculated preparation purity obtained after MACS is considered to be 98.8 ± 0.56, considering that all CD45+/F4/80- cells were eliminated by selective adherence as shown by additional flow cytometry analysis of HSC preparation performed after 24 h of selective adherence (Figure S3A,B).

### 3.3. Cluster of Differentiation 38 Is Expressed on Mouse Hepatic Stellate Cells

**Figure 4:**
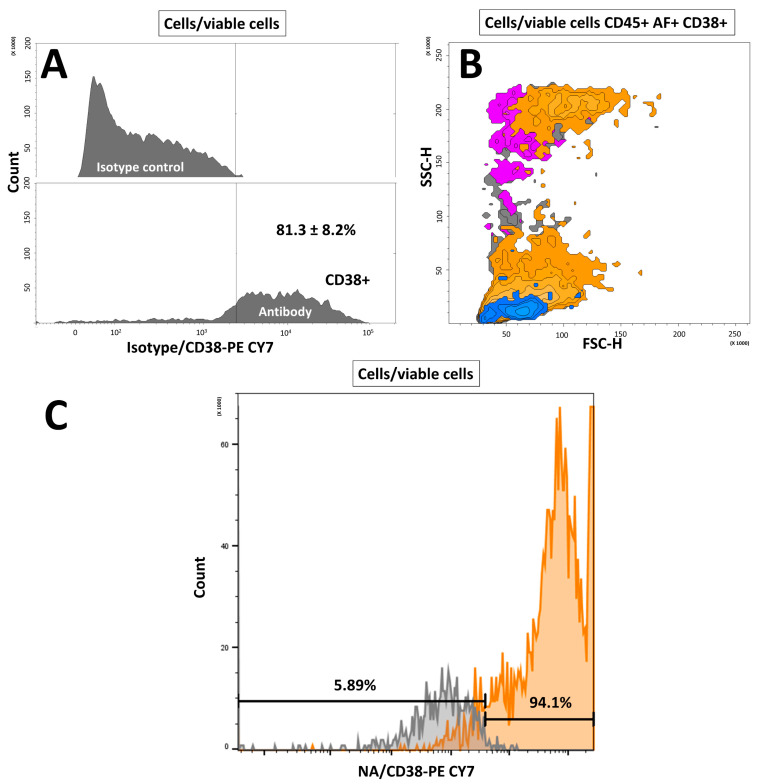
Assessment of CD38 expression on the hepatic stellate cell population sorted by MACS after the density gradient. (**A**) Histogram showing PE CY7 conjugated isotype (upper panel) or PE CY7 conjugated anti-mouse CD38 (lower panel) over cell count. The proportion is expressed as a mean ± SD, *n* = 3. (**B**) Backgating analysis of cell population morphology. Blue: CD45+ cells; violet: violet-induced autofluorescence (AF)+ cells; yellow: CD38+; gray: all viable cells (gate cell/viable cells). SSC: side scatter; FSC: forward scatter. (**C**) CD38 expression of HSC directly sorted from non-parenchymal cells according to their autofluorescence and morphology. Gray: without antibody; orange: CD38-PECY7 staining. *n* = 1, NA: no antibody.

In the isolated cell population, flow cytometry analysis showed that a mean of 81.3 ± 8.2% of living cells expressed CD38 antigen (Figure 4A and Figure S3C,D). From a morphological point of view, we observed that CD38+ cells were distinct from the CD45+ cell population. (Figure 4B). Cells positive for CD45+ were negative for CD38 (Figure S3C,D) and nearly all the AF+ cells expressed CD38 (Figure S3C,D). AF+ cells sorted directly from a crude liver non-parenchymal population also presented a high percentage of CD38+ cells (91.5%), confirming the findings on the MACS-isolated HSC population (Figure 4C). Of note, the autofluorescence of sorted cells was strongly reduced as observed after an additional passage through flow cytometry for analysis (Figure 4C). Such an important reduction in autofluorescence was also observed after selective adherence of HSCs (Figure S3A,B).

**Figure 5:**
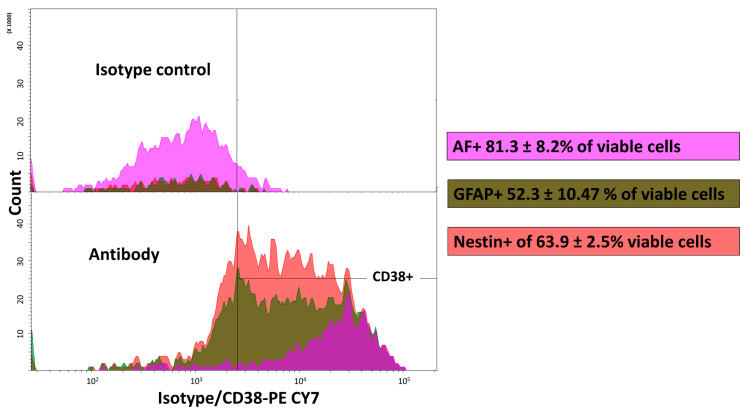
The CD38+ cell population expresses hepatic stellate cell specific intracellular markers GFAP and Nestin. CD38 expression of backgated GFAP+ Nestin+ and violet-induced (AF)+ populations after isotype control (upper panel) and antibody (lower panel) directed against CD38. It appeared that the majority of CD38+ cells were positive for Nestin and GFAP and autofluorescent. Proportions (of viable cells) are expressed as a mean ± SD, *n* = 2.

To determine the characteristics of the CD38+ cells, we performed double staining for surface and intracellular proteins (Figure 5). We observed that Nestin (phycoerythrin-conjugated) and GFAP (Alexa fluor 488-conjugated) were expressed in 63.9 ± 2.5% and 52.3 ± 10.47% of viable cells, respectively. However, the majority of CD38+ cells expressed either Nestin and/or GFAP and all AF+ cells were CD38+ (Figure 5).

## 4. Discussion

HSCs have a strong pathophysiological relevance in liver fibrosis and hepatology. Their cellular functions have mainly been studied in vitro using isolated HSCs from rats and mice where efficient protocols for HSC isolation have been developed [2]. Adequate levels of purity were reached mainly by flow cytometry cell sorting methods based on autofluorescence or granularity that are hallmarks of HSCs [12,29]. Autofluorescence criteria for cell sorting or characterization are, however, restrictive, as HSCs can lose their lipid droplets during the activation process [30], and some HSC subpopulations are poor in vitamin A [17]. Additionally, vitamin A storage is influenced by external factors, such as animal alimentation, age, and genetic background [14].

Further, HSC surface markers have been poorly characterized in mice and only the neurotrophin receptor p75 was used to stain mouse quiescent or activated HSCs on liver sections [31]. Neurotrophin receptor p75 is also found on rat HSCs with tropomyosin receptor kinase C, both constituting the neural crest marker [31,32]. However, tropomyosin receptor kinase C has only been found on quiescent HSCs. Other rat surface markers of quiescent HSC are CD38 [22] and CD133 [33]. PDGF family receptors are expressed by both rat quiescent and activated HSCs [15,34,35]. In addition to these markers, human quiescent HSCs express CD146 [36], CD105 [37], and both quiescent and activated HSCs the mesenchymal marker CD90 [37]. Whether these rat and human markers are also expressed by murine HSCs is not clear. However, numerous rodent intracellular proteins have been reported and proposed to tag quiescent HSCs, such as glial fibrillary acidic protein (GFAP) [38,39], vimentin [40], desmin [40], or activated HSCs, such as vimentin [41], desmin [40], nestin [42], and α smooth muscle actin (α-SMA) [18,43,44,45]. As specific surface markers for further HSC purification by MACS sorting were neither validated in mice nor commercially available, we performed a negative selection of the most frequent contaminant of HSC isolation: macrophages and dendritic cells [21]. Indeed, KCs participates in lipid metabolism [46] and does present lipid droplets [47], or lysosomal inclusions related to their phagocytic activity [48]. Moreover, KC can form doublets with HSCs [49]. This may explain the contamination of macrophages and to a lesser extent dendritic cells (a marginal liver cell population derived from monocytes) in the HSC population obtained by Nycodenz gradient separation [21].

Using the isolation method reported here, the yield of HSCs was higher than the yields reported in the literature for C57BL/6 mice [21]. Moreover, without using autofluorescence cell sorting, we obtained a higher purity with insignificant macrophage contamination compared to existing literature (purity of only 66.8% without autofluorescence sorting) [49]. Paik et al. reported a similar method to purify HSC preparations, but the isolated HSC populations were used for RNA extraction and purity was not described [50]. Moreover, we showed that the function of the cultured HSC was preserved as demonstrated by their capacity to differentiate into myofibroblasts (Figure S2). This method may reasonably be used with other mouse genetic backgrounds.

With the aim to find a potential marker for HSCs in a selected non-parenchymal cell population as obtained after Nycodenz gradient centrifugation, we analyzed CD38 as a marker to characterize all HSC subpopulations. This surface glycoprotein is an ectoenzyme implicated in cyclic ADP ribose formation, and is known to interact with CD31 to transduce inflammatory and proliferating signaling [51,52]. Notably, March et al. demonstrated CD38 implication in HSC activation [22]. In this study, we identified a CD38+/AF+ cell population and a second cell population that was homogenous in size but had heterogeneity in side scatter, extending toward AF+ cells (Figure 4B). This population was CD38+/CD45-/AF- and could correspond to HSCs with a reduced vitamin A content (minority) or HSC subpopulation. Moreover, the HSC cytoskeletal markers Nestin and GFAP, which are the most specific for HSC, were present in the CD38+ cell population. As expected, GFAP and Nestin expression was variable among AF+ and CD45-/AF- cell populations, suggesting a cytoskeletal protein expression reorganization depending on different physiological states of HSCs. Indeed, to the best of our knowledge, GFAP was not reported to be expressed by activated mouse HSCs, as GFAP expression is lost following activation [21,38,39]. Another study suggested that GFAP is expressed in only 70% of HSCs [40]. However, we observed that almost all the CD38+ cell population expressed either GFAP or Nestin, which suggests that these cells are bona fide HSCs. Specificity of CD38 for murine HSCs deserves further investigation, particularly to evaluate whether its expression is maintained when HSCs are isolated from fibrotic livers. Indeed, several authors demonstrated the implication of CD38 in a rodent model of liver chronic inflammation, while these observations probably imply CD38 expressed by macrophages and hepatocytes [53,54].

## 5. Conclusions

We report here an optimized method to isolate HSCs of the C57BL/6 mouse strain, based on a two-step HSC-isolation protocol, avoiding sorting of HSCs through the autofluorescence signal after excitation by UV light. The isolation method resulted in increased yields and high purity compared to methods reported in the literature avoiding autofluorescence cell sorting. The described protocol is advantageous since the process is less time consuming and access to a FACS facility is no longer mandatory for the isolation of pure HSC. Furthermore, we investigated CD38 as a potential HSC surface antigen to identify HSCs in our isolation procedure.

## Figures and Tables

**Table 1:** Cell yield per mouse after HSC Nycodenz separation alone or after additional negative CD11b selection. Nycodenz: yield without CD11b negative selection. CD11b−: HSC enriched fraction (flow through). CD11b+: macrophages (selected cells). SEM: standard error of the mean. Yield variations reflected fluctuations in liver digestion and learning curve of the isolation procedure. *n* = 3 to 7.

	Nycodenz	CD11b−	CD11b+
**Minimum**	220,000	117,500	45,833
**Maximum**	1,146,667	850,000	155,833
**Mean**	451,233	350,595	87,667
**SEM**	174,869	100,773	22,419

## Data Availability

Data will not be shared.

## Supplementary Materials

The following supporting information can be downloaded at https://www.mdpi.com/article/10.3390/cells11091379/s1, Figure S1: Isolated HSC characterization with light microscopy and fluorescence microscopy, Figure S2: Hepatic stellate cell activation, Figure S3: CD38 expression by flowy cytometry, Figure S4: Flowchart of the HSC isolation protocol.

## Abbreviations

AF	violet-induced autofluorescence
α-SMA	α-smooth muscle actin
FACS	fluorescence-activated cell sorting
FBS	fetal bovine serum
GFAP	glial fibrillary acidic protein
HSC	Hepatic stellate cell
IMDM	Iscove’s Modified Dulbecco’s Medium
MACS	magnetic-activated cell sorting
