# Supplementary material for: Optimized Isolation and Characterization of C57BL/6 Mouse Hepatic Stellate Cells
Source: Cells. 2022 Apr 19;11(9):1379. doi: 10.3390/cells11091379 (PMC9102395; doi:10.3390/cells11091379)

Figure S1. Isolated HSC characterization with light microscopy and fluorescence microscopy. (A) After 12 hours of cultured and at high magnification, HSC cytoplasm structure showed numerous cytoplasmic lipid droplets (arrows); (B)Immunofluorescence staining for F4/80 of cell preparations after MACS sorting: retained CD11b+ cells (Kupffer cells) or flow through CD11b- cells (HSC). Blue: Hoechst, green: F4/80. White arrow: a unique macrophage contaminating the HSC preparation; (C and D): Oil Red O staining of HSC droplets confirming their lipid nature. Yellow bar: 50 µm, white bar: 200 µm.

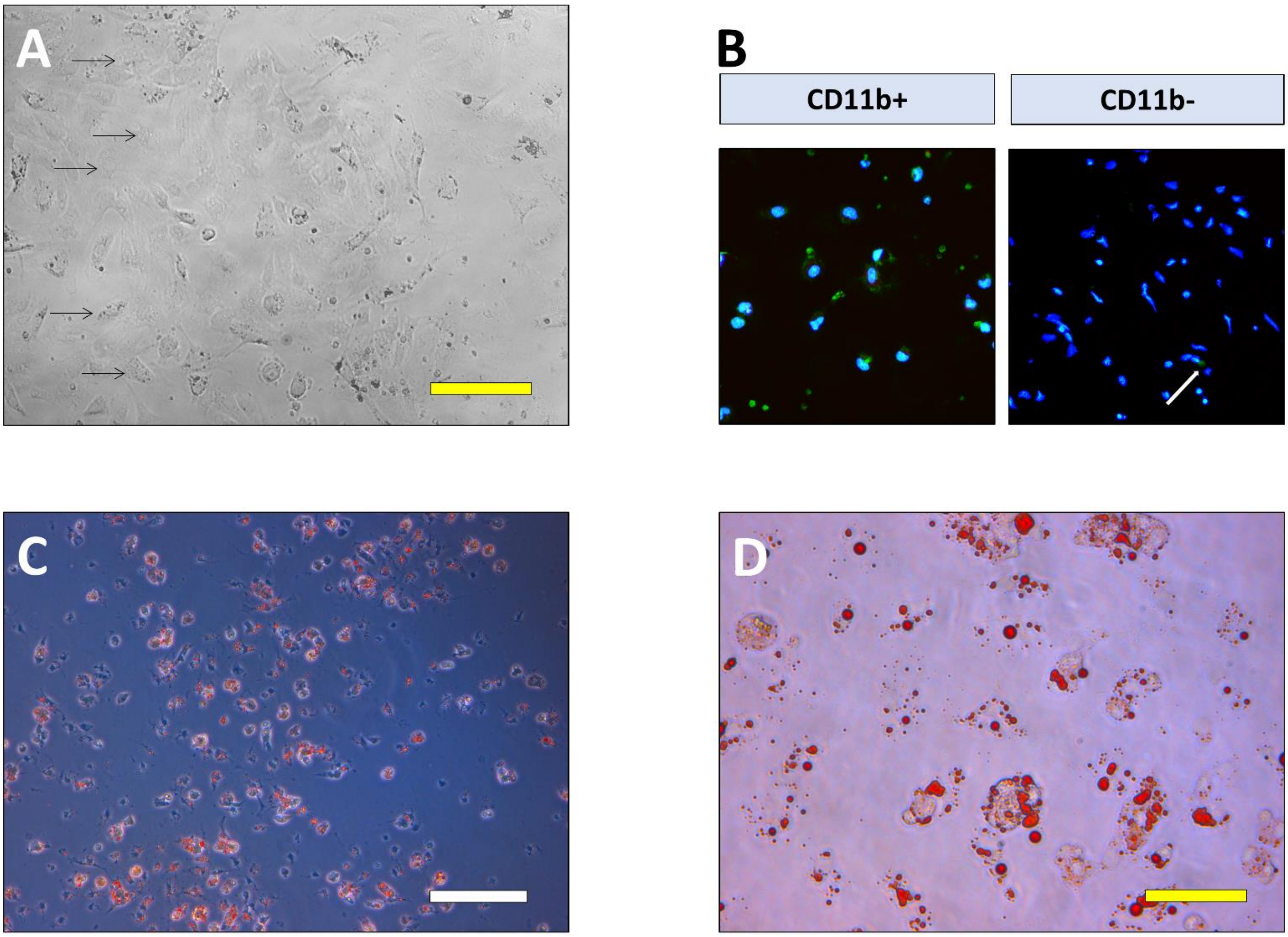

Supplement: Supplementary file 1 [file cells-11-01379-s001.zip › cells-1584918 SM figures/Figure S1.pdf]
