# Supplementary material for: Optimized Isolation and Characterization of C57BL/6 Mouse Hepatic Stellate Cells
Source: Cells. 2022 Apr 19;11(9):1379. doi: 10.3390/cells11091379 (PMC9102395; doi:10.3390/cells11091379)

Figure S2. Hepatic stellate cell activation. Expression of α-smooth muscle (in red, nucleus in blue) after incubation with TGF-β is characteristic of HSC activation and differentiation in myofibroblast.

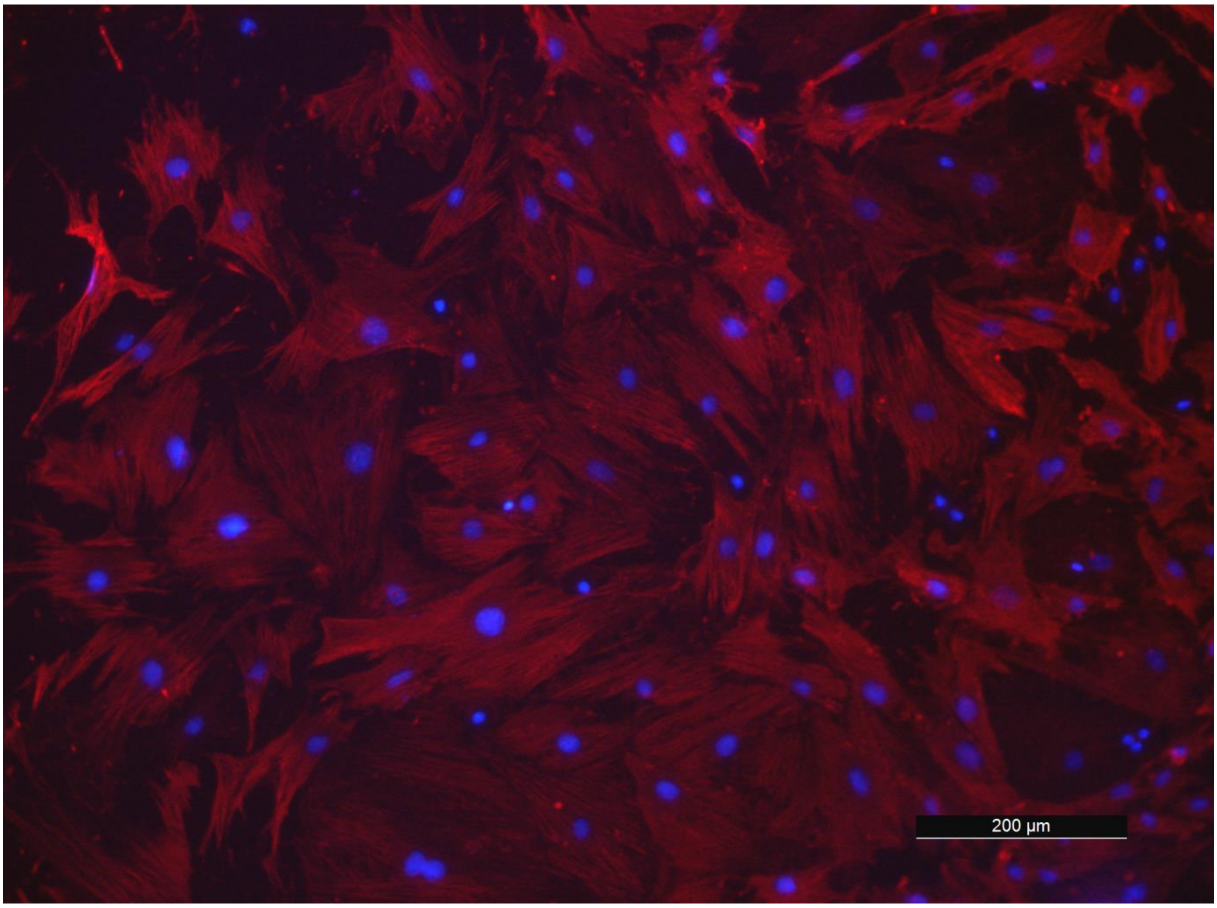

Supplement: Supplementary file 1 [file cells-11-01379-s001.zip › cells-1584918 SM figures/Figure S2.pdf]
