# Supplementary material for: Optimized Isolation and Characterization of C57BL/6 Mouse Hepatic Stellate Cells
Source: Cells. 2022 Apr 19;11(9):1379. doi: 10.3390/cells11091379 (PMC9102395; doi:10.3390/cells11091379)

Figure S3. (A-B) CD38 expression on HSC after 24 h adherence on plastic plates analyzed by flow cytometry using PE CY7-conjugated isotype (A) or anti-mouse CD38 (B) over side scatter. CD38+ cells accounted for 83.7% of viable cells whereas autofluorescent+ (violet) and CD45+ (blue) cells accounted for respectively 8.6% and 5.7%. Overall purity of the preparation was of 92.3% SSC: side scatter, FSC: forward scatter. n=1; (B-C) CD38 expression after incubation with isotype control (B) and antibody (B) and backgating for identified populations. Blue: CD45+ cells, violet: autofluorescence+ cells, gray: CD45-/autofluorescence- cells. n=3.

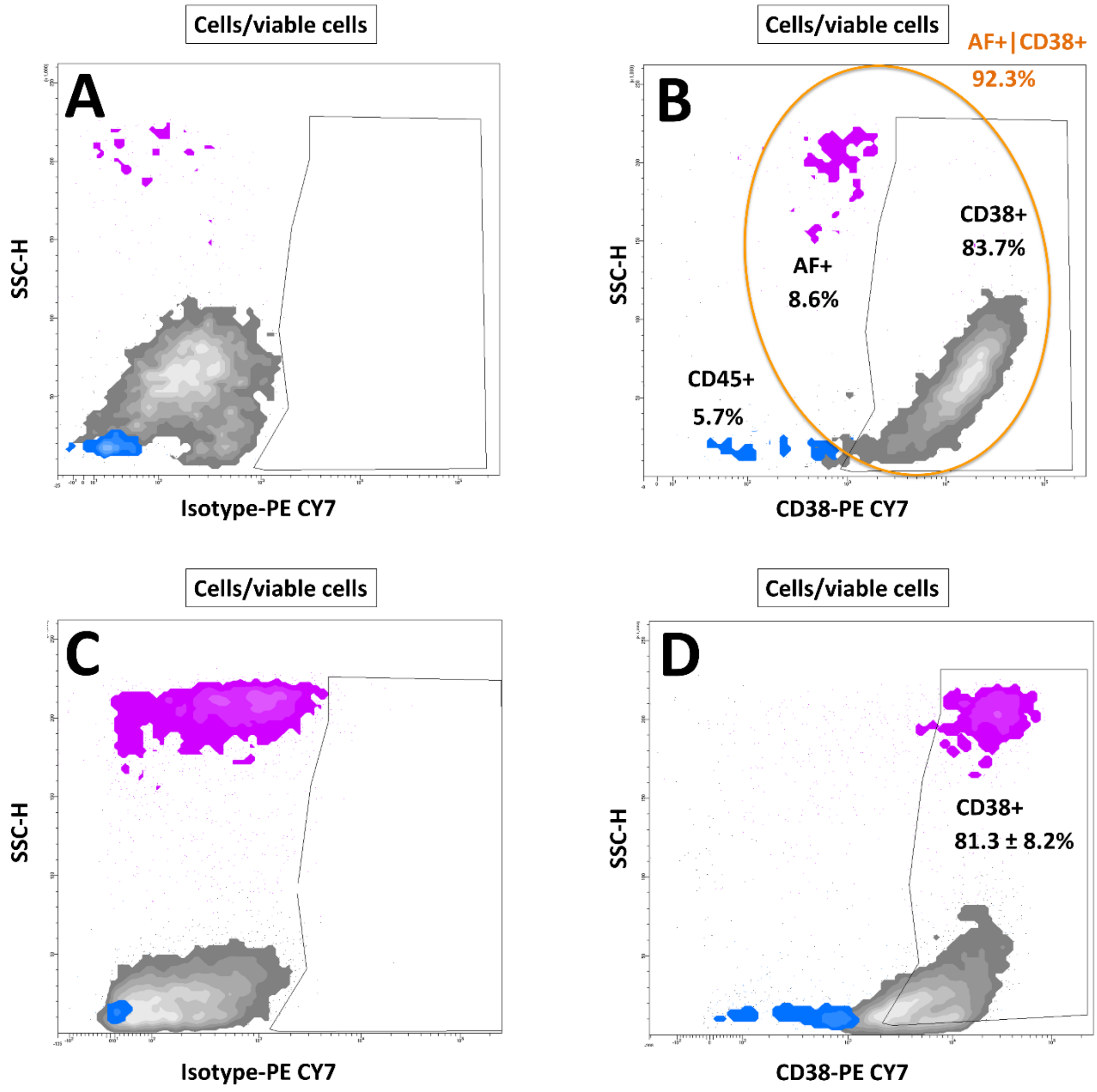

Supplement: Supplementary file 1 [file cells-11-01379-s001.zip › cells-1584918 SM figures/Figure S3.pdf]
