# Supplementary material for: Optimized Isolation and Characterization of C57BL/6 Mouse Hepatic Stellate Cells
Source: Cells. 2022 Apr 19;11(9):1379. doi: 10.3390/cells11091379 (PMC9102395; doi:10.3390/cells11091379)
Supplement: Supplementary file 1 [file cells-11-01379-s001.zip › cells-1584918 SM figures/Figure S4.pdf]

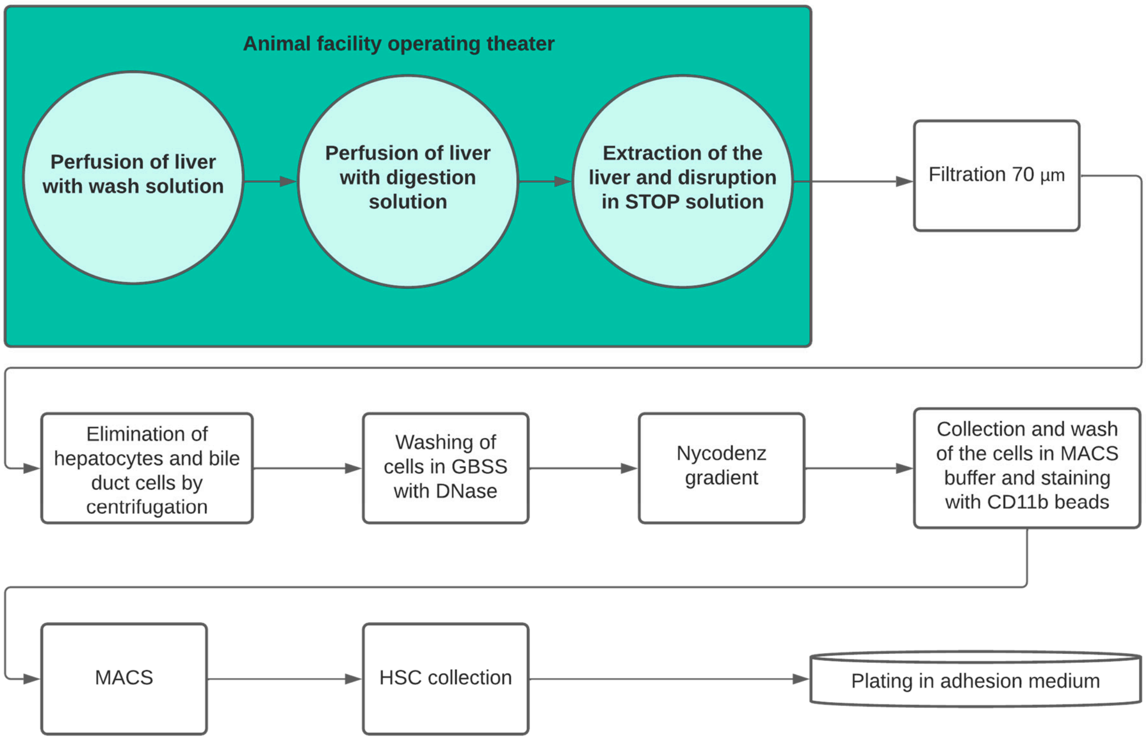

Figure S4. Flowchart of the HSC isolation protocol. MACS: magnetic-activated cell sorting, FACS: fluorescence-activated cell sorting
